# Editorial: Circular RNAs: from anomalies to cellular regulators

**DOI:** 10.3389/fgene.2026.1875072

**Published:** 2026-06-02

**Authors:** Davide Barbagallo, Rosanna Chianese, Francesco Manfrevola

**Affiliations:** 1 Department of Biomedical and Biotechnological Sciences, Section of Biology and Genetics “Giovanni Sichel”, University of Catania, Catania, Italy; 2 Department of Experimental Medicine, University of Campania “Luigi Vanvitelli”, Naples, Italy

**Keywords:** backsplicing, CeRNA networks, circRNA biogenesis, circRNA biomarkers, circRNA diagnostics, circular RNAs

In the traditionally linear landscape of the central dogma, the inherent stability of circular RNAs represents a profound evolutionary masterstroke. Arising from a backsplicing mechanism that covalently closes linear transcripts, these molecules possess a unique structural integrity that shields them from exonucleolytic decay. No longer perceived as passive byproducts of the transcriptional machinery, circRNAs have emerged as pivotal regulatory hubs, utilizing this exceptional stability and functional versatility to exert influence across a vast spectrum of physiological and pathological processes (Conn et al., 2015; Zhang et al., 2016; Errichelli et al., 2017; Kristensen et al., 2019; Chen, 2020; Chioccarelli et al., 2020).

Initially characterized mainly as microRNA sponges, circRNAs are now recognized for their broader functional repertoire, including interactions with RNA-binding proteins (RBPs), regulation of transcription and protein-coding potential (Memczak et al., 2013; Hansen et al., 2013; Chen, 2020; Yang et al., 2017). As a result, circRNAs are increasingly recognized as integral components of gene regulatory networks, with relevant roles in both physiological cell homeostasis and disease pathogenesis (Manfrevola et al., 2020; Chioccarelli et al., 2021; Liu et al., 2022).

The Research Topic ‘Circular RNAs: From Anomalies to Cellular Regulators’ curates a multidisciplinary Research Topic of insights that push the conceptual boundaries of the field. By moving beyond the traditional ceRNA paradigm, these contributions illuminate the role of circRNAs as dynamic architects of gene expression, acting across successive layers of molecular complexity—from broad transcriptomic networks to the precise calibration of specific signalling axes.

In this context, the study by Chang et al. provides compelling evidence of how circRNAs can modulate pathogenic mechanisms, reinforcing the view of circRNAs as upstream modulators of complex disease-related pathways. Focusing on intrauterine adhesion (IUA), a condition characterized by endometrial fibrosis, the authors combined transcriptomic profiling with integrative bioinformatic analyses to identify differentially expressed circRNAs and to provide circRNA–miRNA–mRNA regulatory networks (ceRNETs) associated with extracellular matrix remodelling. Within these, hsa_circ_0000994 and hsa_circ_0000439 emerged as key regulatory actors, as supported by the silencing of hsa_circ_0000994 that resulted in a marked reduction of fibrosis-related markers. In line with these results, pathway enrichment analyses performed by Chang and co-authors revealed the activation of major signalling cascades including NF-κB and Notch, both critically implicated in inflammation and fibrosis, thereby linking circRNA dysregulation to well-established pathogenic pathway.

Shi et al. further refine this regulatory concept by describing a circRNA-mediated axis in eosinophilic chronic rhinosinusitis (ECRS), a highly treatment-resistant inflammatory condition. By comparing nasal polyp tissues from ECRS patients with non-inflammatory controls, the authors identified a distinct expression pattern characterized by upregulation of circRNA_0021727 and its downstream target ADAM12, alongside downregulation of miRNA_145_5p. These findings support the existence of a circRNA_0021727–miRNA_145_5p–ADAM12 regulatory axis, in which the circRNA acts upstream by modulating miRNA availability and, consequently, target gene expression. Beyond molecular interactions, this axis is functionally linked to inflammatory responses through the modulation of key mediators including GM-CSF, EOTAXIN and MUC5AC.

Consistent with this view, the reviews by Chen et al., Guan et al., and Bernal-Vázquez et al. highlight the involvement of circRNAs in shared stress-response pathways including inflammation, apoptosis, oxidative stress and metabolic dysfunctions.

Chen et al. situate circRNAs within a spectrum of pancreatic diseases such as acute pancreatitis (AP), chronic pancreatitis (CP) and pancreatic cancer (PC). In this context, circRNAs emerge as modulators of inflammatory and cell death pathways in AP, displaying context-dependent functions across disease stages. Notably, same circRNAs may exert opposing effects depending on the pathological context, from promoting inflammation in acute injury to supporting survival and tumor progression in cancer. This functional plasticity highlights the involvement of circRNAs in shared regulatory axes linking inflammation, metabolic reprogramming and disease progression. Importantly, the authors propose that circRNAs may regulate key pathways such as TGF-β signalling, pancreatic stellate cell activation and extracellular matrix remodelling, thus suggesting a unifying role in chronic tissue damage. Beyond mechanistic insights, Chen et al. emphasize the translational of circRNAs as non-invasive biomarkers and potential therapeutic targets.

Guan et al. extend this framework to the cardiovascular-metabolic context by examining the role of circRNAs in diabetic cardiomyopathy (DCM), a complex disorder characterized by the interplay of metabolic dysregulation, oxidative stress, fibrosis and cardiomyocyte death. Also in this case, the review highlights how under similar pathological conditions different circRNAs may exert opposing effects as: circFOXP1 exerts cardioprotective functions by attenuating oxidative stress and apoptosis, whereas circHIPK3 promotes mitochondrial dysfunction and ROS production aggravating disease progression. Beyond mechanistic insights, the review underscores potential clinical translation. As matter of fact, the authors support for some circRNAs, such as CDR1as, CACR and circHIPK3, the potential relevance as non-invasive biomarkers and therapeutic targets in DCM.

Bernal-Vázquez et al. expand this Research Topic to obesity-associated diseases, positioning circRNAs as central modulators of metabolic dysfunction and chronic inflammation across multiple organs. In obesity-associated cardiovascular diseases, circ-Foxo3 promotes cardiac senescence by sequestering anti-stress and anti-senescent proteins (including ID-1, E2F1, FAK, and HIF1α), whereas circANRIL exerts a protective effect in atherosclerosis by regulating vascular smooth muscle cell and macrophage proliferation. Their detectability in circulation and disease-specific expression profiles further support their potential as biomarkers for cardiovascular risk stratification in obese patients. The authors further amplify the role of circRNAs in obesity-associated metabolic disorders. Specific circRNAs such as circHIPK3, CDR1as and circGlis3 are implicated in the regulation of pancreatic β-cell function and insulin secretion, while circSCD1 is in hepatic lipid accumulation and progression toward non-alcoholic steatohepatitis.

In obesity-linked cancers, circRNAs participate in the intersection between metabolic reprogramming, inflammation and tumor progression. CircSAMD4A links adipocyte differentiation and lipid accumulation to systemic metabolic alterations, while circACC1 and circ-MAT2B drive tumor metabolic flexibility by regulating AMPK signalling and HIF-1α-mediated glycolysis, respectively. In parallel, inflammation-driven circMRCKa induced by tumor-associated macrophages further connect immune signalling to cancer cell metabolic adaptation. Overall, these findings illustrate circRNAs as mediators of intercellular communication within the obesity-altered microenvironment. Despite strong correlative evidence, functional validation in integrated models and longitudinal studies remains limited. Nevertheless, circRNAs such as circHIPK3, CDR1as, circACC1, circANRIL, and circ-Foxo3 consistently emerge as central nodes across these interconnected disease networks.

Collectively, the contributions within this Research Topic provide compelling evidence for a paradigm shift, firmly establishing circRNAs as sophisticated molecular protagonists rather than mere transcriptional relics. Across a diverse pathological landscape, these molecules have emerged as pivotal integrators of metabolic, inflammatory and tumor signalling. Consequently, circRNAs transcend their utility as diagnostic biomarkers; they represent a frontier of upstream therapeutic intervention, necessitating a deeper integration into the frameworks of fundamental cell biology and the burgeoning field of precision medicine.
